# A Study of Traveller Horse Owners’ Attitudes to Horse Care and Welfare Using an Equine Body Condition Scoring System

**DOI:** 10.3390/ani9040162

**Published:** 2019-04-12

**Authors:** Marie Rowland, Tamsin Coombs, Melanie Connor

**Affiliations:** 1 Royal (Dick) School of Veterinary Studies, Easter Bush, Midlothian EH25 9RG, UK; m.connor@irri.org; 2 SRUC, Roslin Institute, Easter Bush, Midlothian EH25 9RG, UK; Tamsin.coombs@sruc.ac.uk

**Keywords:** horses, horse welfare, attitudes, Travellers, body condition scoring

## Abstract

**Simple Summary:**

Irish Travellers are an indigenous ethnic minority group. Horses are an important part of Travellers’ lives, with horse ownership considered one of the last links to their nomadic way of life. Travellers keep, breed, and sell horses. Trotting and sulky racing are popular recreational activities. Traveller horses are often viewed as having poor health and welfare by the equine industry and the media although there is little data to support these views. This study aimed to investigate Traveller horse owners’ attitudes to horse care and welfare by using in-depth interviews and body condition scoring, a well-known tool used to detect welfare related conditions, to investigate Travellers’ understanding of horse welfare. The ease of use and accuracy of the body condition scoring tool as applied by Traveller horse owners was assessed. Results indicate that they had a good understanding of a horse’s natural behaviour, which is reflected in their management practices. A major barrier to improved horse welfare cited was land availability. Travellers were found to be able to use the scoring tool easily although further training is recommended. This study provides us with an understanding of how Travellers’ manage their horses. Further research is recommended with a larger sample group.

**Abstract:**

Traveller horses are often perceived to be exposed to poor welfare due to Travellers’ traditional way of horsemanship. However, few studies have investigated Traveller horse welfare. Hence, the present study aims to explore Traveller horse owners’ attitudes to horse care and welfare. Semi-structured interviews and discussion groups examined 14 Irish Traveller horse owners’ attitudes and approach to horse ownership. Additionally, a body condition scoring (BCS) instrument was assessed for its accuracy and ease of use when applied by Traveller horse owners. Additionally, the BCS system was used to assess 18 horses. Results show that Travellers have a good understanding of horses’ natural behaviours and environment, which is reflected in their management practices. However, barriers to improved welfare are land availability, since landowners are often reluctant to lease to Travellers, and the impoundment of horses as a consequence of fly grazing, under the Control of Horses Act 1996 (Ireland). Furthermore, Travellers regarded the BCS as a useful tool, but would require training to apply the scoring successfully. The results suggest that attitudes and management practices are favourable, but Travellers have limited means to overcome barriers. Therefore, it is necessary to increase capacity building and assist with the acquisition of land.

## 1. Introduction

The estimated horse (*Equus caballus*) population in Ireland is 160,000 [1] with horses fulfilling many functions, from work to recreation. The economic recession in 2008 resulted in a significant increase in the number of unwanted horses [2]. Consequently, equine welfare has become a greater concern for both scientists and society, alike [3]. Welfare concerns exist in all sectors of the horse industry in Ireland. Collins et al. [4] explored the perceptions of equine welfare issues using vignettes with representatives of the Traveller community involved in the Policy Delphi process. Respondents and interviewees identified Traveller horse owners as specific contributors to compromised equine welfare during the Policy Delphi process. In a follow up study, Traveller men were included in two focus group discussions with the aim of gaining a better understanding of the role of the horse in Traveller culture [5]. While recognising that there may be some horse welfare concerns within the Traveller community, the authors concluded that welfare concerns are evident within a subsection of all horse owning communities [5]. A review of the literature found no recorded information on the population of Traveller/Gypsy horses in Ireland and the extent of Traveller horse welfare issues, if any, has yet to be established.

Travellers are defined ‘as people with a shared history, culture and traditions, including, historically, a nomadic way of life on the island of Ireland’ [6] and account for at least 0.5% of the population of Ireland [7]. Travellers are still seen as outsiders in Irish society [8] and like other Gypsy communities, Irish Travellers have endured discrimination and hostility with their experiences described as one of social and cultural exclusion [9]. Today, Travellers are still labelled as ‘criminals’, ‘asocial’, and ‘deviants’ [10]. Remarkably, Irish Travellers were only recognised as an ethnic group in Ireland in March 2017 [11].

Travellers have always regarded horses as central to their lives [12]. The original Traveller horses were coloured cobs and the vanners, used to pull their wagons. As Travellers’ lifestyle became more mechanised, the key roles of horses have changed as dependence on horses for movement has declined. Today, Travellers consider horse ownership as one of the last links to the Traveller nomadic way of life. They keep, breed, and sell horses and participate in traditional horse fairs (e.g., Ballinasloe in Ireland and Appleby in England). Furthermore, trotting, harness racing, and sulky riding are popular recreational activities, which have been traditions for generations [12]. The limited literature indicates that common practices, such as fly-grazing, straying and abandoned animals, and indiscriminate breeding, compromise Traveller horse welfare [13]. The Control of Horses Act [14] was introduced in Ireland following public concerns about wandering horses, property damage, and neglect of horses. Proposals on the implementation of the act in Ireland from a governmental advisory group did not include representatives from the Traveller community or Traveller organisations, thus from the outset, the needs and challenges of Traveller horse ownership were not considered. The Control of Horses Act prevents horses from straying onto public or private property without the permission of the land owner, with the prohibited horse owner liable for any damage caused. Local authorities are responsible for enforcement of the act, often resulting in the impoundment of horses. This act makes it extremely difficult for Travellers to support their tradition of horse ownership given the shortage of land ownership, poor accommodation status, and discrimination [12]. Difficulty in leasing land was cited in a feasibility study into the establishment of a horse care project [15] as landowners are often hesitant to lease to Travellers [16], perhaps because of preconceptions often based on negative stereotyping as previously discussed [10]. The Irish Traveller Movement (ITM) argues that Traveller culture and heritage is being depleted as a consequence of this act [12]

It has been suggested that in order to improve equine welfare, it is necessary to educate owners on horse care and welfare [17], with Collins stating the need for education and training across all horse ownership sectors [5]. While there are various educational and materials focusing on general horse ownership [18], novel initiatives are required to address the needs of those often considered hard to reach, such as Traveller horse owners [5]. Human public health studies have shown that culturally sensitive health education had a positive impact on health in ethnic minority groups [19]. In the last decade, the Irish Horse Welfare Trust has delivered various education programmes to young disadvantaged youths, including Travellers, while at the same time promoting horse welfare [20]. An introduction to farrier skills was completed by Traveller horse owners at Shannon Horse Project (2014) and a programme on Animal Welfare Education was delivered by Travellers of North Cork to over 20 Traveller men (2018). A number of welfare agencies operate an education stand at the annual Appleby Horse Fair, England, which is one of the largest gatherings of Travellers and Gypsies in Europe. The ITM recommends horse projects designed in consultation with Traveller horse owners to help address the specific issues encountered by this group [12]. Horse projects offer resource and animal based support to Traveller horse owners and Fettercairn’s Youth Horse Project and the Cherry Orchard Equine Project, both in Dublin, are cited as good models since participants of these projects develop both personal and practical skills [21]. Collins et al. (2011) claims that in general, social horse projects, while commendable in their attempts to both engage with urban horse owners and improve local horse culture, do not result in tangible nor long term improved human wellbeing or horse welfare [22]. Nevertheless, he refers to one project in his study as having had a positive impact on local horse culture. The Cherry Orchard Equine Project acquired grazing land, indoor and outdoor riding facilities, and stables and now owns its own teaching horses. They provide training in both riding and general horse care to local people and in 2010, around 600 people attended weekly courses intended to improve knowledge and skills and also provide a pathway to careers in the equine industry. Unfortunately, it appears that this programme is in the minority so it is clear that the continuing evaluation of these interventions is necessary to determine whether these programmes are efficient and effective and to inform the planning and content of further courses and interventions.

### 1.1. Welfare Assessment

Traditionally, the prevention of animal suffering was at the centre of welfare assessment with assessment protocols based upon the five freedoms [23] and comprising of a number of resource-based measures [24,25]. However, rather than assess one facet of an animal’s state, such as its physical state alone, welfare assessments now adopt a more holistic approach [26]. Thus, an effective assessment protocol should incorporate a holistic approach to animal ‘welfare’ as well as the aims and objectives of the welfare assessment. Currently, there are various tools and indicators available to measure equine welfare with both animal and resource-based measures [27] and human attitudes and behaviour [28] used in the measurement process. Most importantly, measures need to be valid, reliable, and applicable to diverse situations and assessors [29]. 

#### Evaluation of Equine Welfare

Body condition scoring (BCS), an animal based measure, is used to assess body condition in many species. Using a numerical scoring system, the BCS tool estimates the amount of fat on a horse’s body through visual appraisal and palpable assessment [30]. In the application of BCS, key body regions are identified; the neck, back, ribs, and rump of a horse and used to measure adiposity with different scales available to calculate an overall score [31,32]. All scales follow the same principle in that a high score indicates an obese horse and a lower score refers to an emaciated horse. These scales are simple and easy to learn, but are very subjective as ratings may differ between scorers using the same scale [33]. Wyse et al. [34] found that there was only 50% agreement between respondents’ and researchers’ BCS in their study on the prevalence of obesity in riding horses in Scotland. Factors found to affect the ability to BCS accurately include breed and age of horse [32], horse type [35], hair thickness [36], and owner/carer experience [37].

In evaluating equine welfare, human attitudes to animals also need to be included in the welfare assessment system as human attitudes are found to influence animal welfare [38]. In fact, Hemsworth et al. [39] found that negative attitudes towards animals correlated with poorer care and management practices. While few studies exist on the attitudes of responsible or neglectful horse owners [3], there are a number of studies regarding people’s attitudes towards other types of animals. Studies on farm animals found that a stockperson’s attitude and behaviour towards an animal can either have a positive or negative effect on that animal’s health and welfare [40,41]. Horse owner attributes too can have an influence an animal’s health and welfare status. Attributes, such as lack of horse management knowledge and lack of commitment to horse ownership, are also perceived to influence welfare [42,43].

### 1.2. Rationale for Present Study

This research is important because at a time when equine welfare is high profile and with the welfare of Traveller’s horses perceived as being a major area of concern, to the best of our knowledge, Traveller horse owners’ attitudes to horse care and welfare have not been examined in scientific research. Qualitative research methods were used in order to gain information on experience, meaning, and perspective from the standpoint of the Travellers themselves. It allowed the investigation of knowledge, beliefs, attitudes, and concepts of normative behaviour, thus providing a greater understanding of Irish Traveller horse ownership and management. 

## 2. Materials and Methods

A multi-method approach of qualitative interviewing and quantitative body condition scoring was used to examine the knowledge and attitudes to horse welfare of Traveller horse owners. Semi-structured interviews and group discussions were used with these formats and allowed participants to express their views and beliefs in their own words. Participation in either the interview or focus groups depended on the participant’s personal preference. Body condition was assessed, based upon Carroll and Huntington’s scoring tool [32], and was used as a medium to introduce the study to the group since it has been reported to be easy to use and it is also the most commonly used tool in the UK and Ireland.

### 2.1. Recruitment and Participants 

In total, 14 Traveller men who were either past or current horse owners with an age range of 18 to 54 years were recruited for both parts of this study. They were recruited through the means of direct conversation and all participants were members of a horse project, which was set up in 2010 in the West of Ireland (due to data protection and anonymity the name of the project cannot be disclosed). Following negotiations on the leasing of land between Travellers and the local authority, the project resulted is a 33-acre site, including a sand arena and a number of stables. The lead author was the sole interviewer involved in the development of this project and therefore all participants were already familiar with her and willing to accept a female interviewer for what is seen as a male dominated activity in Traveller culture. The horses that were used for BCS assessment were owned by participants and were housed at the Traveller Horse Project and at land nearby. Recruitment and data collection occurred between September 2013 and December 2013.

### 2.2. Interview/Discussion Questions Design

Semi-structured interviews were based on a series of open-ended questions and the questions that were used are presented in Table 1. Since semi-structured interviews and discussion groups were used, questions were only asked when participants did not talk about the topic by themselves. 

Furthermore, the wording of the questions presented in Table 1 could vary to accommodate participants’ level of understanding and to act when needed to repeat and/or re-phrase questions. Probing questions were used where appropriate to explore responses more thoroughly and provide a deeper understanding of the subject. In this study, probing questions were used to evaluate the relevance of statements, to check for understanding and clarification of situations, to provide more detail and information, to seek examples when participants were vague with their responses and to understand a participant’s feelings. Probing questions were open-ended and most often began with ‘what’, ‘when’, ‘where’, ‘why’, and ‘how’. The interview was pre-tested with Traveller horse owners and based on feedback, the wording was changed on four of the interview questions so that they would be more clearly understood. All interviews and group discussions were transcribed.

### 2.3. Procedure

Participants were given a verbal summary of the study aims and activities and an explanation of Carrol and Huntington’s BCS system [32] was presented. Data was collected from three semi-structured interviews and four group discussions consisting of between two and four participants. Interviews and group discussions were conducted face-to-face and lasted 45 to 60 minutes. Brief notes were taken in a discrete way, ensuring that the flow of conversation was not interrupted, and when consent was given interviews/group discussions were voice recorded on a Lenovo tablet. In appreciation of participants’ participation, the Horse Project was presented with equine first aid kits at the end of the study.

### 2.4. Body Condition Scoring Assessment

The practical application of BCS followed immediately after the interviews/discussions. The BCS chart [32] was revised for this study (Table 2), taking into account the literacy levels of Travellers, which is reported to be lower than that of the general population in Ireland [44]. An averaging system, allowing for half-point responses, was applied to the BCS system [45]. This system entails separately scoring the neck, middle, and quarters of the horse and then calculating the mean of the three scores for an overall body condition score. As horses accumulate their weight in different areas, this system is considered a more accurate assessment [46]. Two different scoring sheets to record body condition scores were devised, one for the researcher and the other for participants. For each horse, age, sex, and breed was recorded.

On the morning prior to the task commencing, horses were numbered and body conditions scored by the researcher. Participants were given a copy of the revised BCS chart and a demonstration of BCS by the researcher. Horses selected for BCS were randomly allocated to participants to be assessed. Horses were assessed independently by each participant and scores recorded. Participants were not given any guidance during the BCS assessment and were unaware of the scores of the other participants’ assessments. All participants scored a minimum of two horses, with a maximum of 14 horses scored by one individual. Twelve participants scored at least one of their own horses with two participants not owning horses at the time of assessment. The variation in the number of horses assessed was due to certain participants’ time constraints and other commitments on the day. 

### 2.5. Ethical Approval 

This study was approved by the Student Survey Overview Group, University of Edinburgh (Ref no. SSOG/15/99).

### 2.6. Data Analysis

#### 2.6.1. Interview Analysis

To obtain key information and define themes present in the transcripts, coding and thematic analysis was conducted using NVIVO 10 (QSR International, Melbourne, Australia) a software programme designed for qualitative data analysis. Transcripts were coded using a defined coding scheme for attitudes, values, meanings, beliefs, experiences, and knowledge characteristics. The primary coding terms were determined by the topics covered in the interview questions. In-depth readings of the transcripts and further analysis revealed key concepts that consistently arose and were relevant to the project’s aims. Participants’ responses were examined and areas of agreement and difference on issues were identified. Responses from the total number of participants (*n* = 14) were analysed together. As not all participants discussed each question, the proportion of the sample who contributed to specific questions is presented in the results. The final stage of the analysis was to formulate themes with the final section of themes grounded in the study aims and in the views and experiences of the participants. 

#### 2.6.2. BCS Analysis

For the purposes of analysis, the original half point scores were converted to whole numbers and the overall score was calculated as the median of the neck, middle, and hindquarters before being converted to a whole number. The statistical package, Minitab 17, was used to conduct descriptive statistics and Cohen’s kappa. Cohen’s kappa was used to determine the level of agreement between the researcher’s scores and participants’ scores when assessing horses’ body condition. Level of agreement was assessed using Viera and Garrett’s classification system as follows: Kappa agreement <0 less than chance agreement, 0.01–0.20 slight agreement, 0.21–0.40 fair agreement, 0.41–0.60 moderate agreement, 0.61–0.80 substantial agreement, and 0.81–0.99 almost perfect agreement [47] Analysis included the level of agreement on overall scores using the median and each section scored, i.e., neck, middle, and hindquarters. 

## 3. Results

### 3.1. Demographics

All participants (*n* = 14) were Irish Travellers and lived in the West of Ireland. Participants’ age ranged from 18 to 54 years, with a mean age of 29.5 years (SD = 10.84). Body condition was assessed in 18 horses and ponies with an age range of 1.5 to 28 years and a mean of 8.25 years (SD = 5.97). Most animals (*n* = 16, 88.8%) were female (mares) with the remaining two horses being uncastrated males (stallions). Most of the horses were full or part-bred Standardbred (*n* = 10, 55.5%) with the remaining animals of Thoroughbred (*n* = 3 (16.6%), mixed breeding (*n* = 2, 11.1%), or described as miniature ponies (*n* = 3, 16.6%). 

### 3.2. Results of the Interviews/Group Discussions

In total, seven themes were identified. Results are presented according to finalised themes. Participants’ quotes are in italics.

#### 3.2.1. Theme 1: Perception of Body Condition Scoring

From the outset, all participants indicated that they were unfamiliar with the BCS tool. References to BCS procedure were predominantly positive with participants stating that *‘looking*’ at the horse was important while a single participant claimed that the palpation of fat was a necessary feature of assessment because *‘you wouldn’t know fat by looking at it*’. There were many references to the benefits of using BCS. Participants referred to these as benefits for health and wellbeing and reported that it is especially important to assess mares in foal. Further participants believed BCS could help identify feeding patterns, obesity, or may expose bullying. Participants claimed that they would use the BCS tool, with some of the participants agreeing with the anatomical descriptors of BCS. However, it was suggested that other areas of the body should also be included, such as the chest and hips, as they believed that fat accumulates in these areas. When asked for the difference between fat and muscle, participants were able to explain the difference, *‘Well, you would know by the tone of it. You would know, there’s a difference’* and *‘Muscle would be solid, fat isn’t, there is a difference’.* It was generally agreed that the BCS tool was applicable to all horses with references made to the need to understand how to use it. As a common view, participants agreed that all horse owners should use BCS, but differed in their responses to the frequency of use, *‘Every couple of weeks really, to keep on top of things’* as opposed to *‘At the beginning of the winter’*. Body condition was also believed to be a good guide of the suitability of diet, *‘Yeah, if they are fed, their condition will be good’.*

#### 3.2.2. Theme 2: Horse ‘Health’

When asked ‘Can you tell if a horse is healthy by looking at it?’, participants reported that they could with one participant disagreeing. References to a healthy horse included body condition, form, energy, weight, and coat and teeth condition. When asked ‘how can you tell if a horse is sick’, participants cited behaviours, such as *‘head down’*, *‘standing in the corner of the field’*, *‘rolling’*, *‘not feeding’*, and *‘not doing things that you would normally see the horse doing*’. When asked what these ‘things’ are, the participant stated that horse would *‘not be eating, grazing or happy in himself’.*

De-worming was considered crucial for health by over half of the participants with varying references to frequency, *‘Vital, isn’t it. Their health depends on it’*. A minority of respondents suggested three to four times a year with one adding that it depends on the horse and another advocating for dosing of all horses for prevention. De-worming of all horses to prevent transmission was considered important for group health. Participants demonstrated that they understood the difference between a grass belly and obesity; *‘A grass belly, any horse that is out grazing four or five months will come in with a grass belly as being obese is constantly’.* One respondent was of the opinion that a grass belly would recede when taken off grass. All of those who responded agreed that an understanding of body condition would help prevent future health problems. Health problems, such as over/underweight, laminitis, worms, and parasites, were cited, *‘Condition is crucial for her health’.*

#### 3.2.3. Theme 3: Views on ‘Exercise’

It was commonly perceived by participants that exercise is important for horses and that exercise is natural to outdoor horses. Participants expressed the view that structured exercise is necessary for both physical and mental health for stabled horses, ‘*But if you have him confined in a stable, he needs, it’s crucially important. His bones, his legs would go wrong and he would go demented. He needs a workout, yeah’*. Ringing, commonly known as lunging and sulky/cart trotting, were identified as key forms of exercise. *‘A bit of ringing, have it at the end of a rope & ring it around, maybe a half hour outside’.* A discussion further revealed that some participants take their horses swimming. Participants also considered music and ball playing beneficial to a horse’s mental wellbeing*, ‘Or a radio, horses love a radio. A lot of people play a tape recorder or radio for the horse, it’s good for the mind’.*

There was a degree of variety in participants’ responses to the appropriate age to commence exercise with the majority stating at two years although one participant claimed that it may not be right at this age. The reason cited that age differs for Traveller horses was that their horses need to be introduced to the road and traffic at an early age as the horses are used for trotting and sulky racing. Behaviour management was another reason proposed for handling at a young age with younger horses easier to handle, ‘*For Travellers, horses see the road from a year or so, so that they get used to the traffic and that’.*

#### 3.2.4. Theme 4: Perception of ‘Social Interaction’

Participants expressed the view that horses form bonds with their owners with two claiming that they can identify their owner’s voices. Participants believe that bonds exist within herd mates, with one stating that these bonds have a protective element, especially for mares with foals, *‘And to protect their young, they would die to help their own’*. A consistent viewpoint was that horses develop friendships within the herd, *‘If you had horses in a field, you could have 20 but you would get three that would be best friends’.* There was a strong consensus that bullying exists, with some horses more vulnerable than others. Inadequate land size and introduction into herds were reasons quoted, *‘Yeah, if there wasn’t enough ground, they would get bullied’.* The link between bullying and health was reported with claims that it needs to be monitored and action taken if necessary expressed. 

#### 3.2.5. Theme 5: ‘Existence of Sentience’ in Horses

Participants overwhelmingly stated that horses have feelings, with the belief that horses are intelligent also reported, *‘By God, they do, no question and very, very intelligent, believe it or not’.* Feelings cited included fear, pain, and loneliness. The view that horse ownership has a positive effect on participants’ own mental health was expressed by various participants, *‘I trot for my own mind’* and *‘They are nice to have and they are good for the mind’.*

#### 3.2.6. Theme 6: The Horse’s ‘Natural Environment’

There was unanimity across respondents that it is natural for horses to live in groups. Company, to prevent loneliness and for warmth, were reasons given for this preference, *‘Our horses have company all year round’* and *‘Like any animal they don’t want to be alone’.* One sentiment expressed frequently was the importance of keeping horses outdoors, believing it is natural, *‘I prefer if they are outside, horses are hardy they like company’.* Constant access to grazing with additional feed when necessary was the principal view of participants, *‘Yes, they need to be able to go out and graze. It’s natural to them*’. Further participants were of the opinion that continuous grazing could lead to weight gain so should be monitored. Some participants described how their feeding patterns are seasonal with horses fattened up before the winter months when less fodder is available, *‘We feed up the horses for the winter and then they will reduce some of that fat’*. There were a number of coded references on the use of rugs. Participants varied on their responses with several participants suggesting that rugs are only necessary in very harsh weather or for sick and vulnerable horses, *‘It doesn’t do the horse any favours‘,* ‘*If the weather is ok and there is shelter, they don’t need them’.* The view that horses require rugs was expressed by a minority of participants. Benefits of rug-use cited were *‘no water scabs’* and ‘*to keep his body’*. One participant was adamant that horses only need rugs in exceptional cases and continued to describe the public’s expectation of horses needing rugs. Frequent references were made to the practice of ‘*looking*’ at the horse and how this was considered a natural activity in horse management, ‘*But visually you would know if she is looking poorly or not herself’.*

#### 3.2.7. Theme 7: The Horse’s ‘Welfare’

Good health was almost universally regarded as an indicator for good welfare with over half of those who responded specifying that good health indicates that horses are being ‘looked after’. All participants agreed that neglect exists within all sectors, including within their own culture, *‘In fairness, Travellers do cause a bit themselves’.* One participant believed that neglect does not exist in the racing sector, but then said that ‘*they can use the whip a bit too much at times’* and agreed that this was a welfare issue. He also stated that there was a relationship between welfare and economics. Another view was that the ‘*bigger guys*’ are less likely to be investigated about their practices. One participant offered an alternative view, claiming that people are inherently good with their horses. Participants differed in their opinions over whether horse breeding should continue in the current climate; some participants disagreed while others agreed, if resources are available. Some participants said it should be controlled while one participant agreed with breeding, *‘If you can look after them, it’s ok but if you can’t you should not’.* Participants consistently made the same points in relation to difficulties experienced and their implications. The majority of participants who responded cited leasing of land as the greatest difficulty faced by Travellers, *‘What is the biggest problem for Travellers is land*’. The value of the Horse Project was highlighted in helping combat this issue. *‘It’s good to have the land down below’.*

### 3.3. Results of Body Condition Scoring

The second aim of this study was to assess the BCS tool for accuracy and ease of use. Results are shown in Table 3. For the overall BCS score, 66.67% of participants scored their horses in accordance to the researcher, indicating a moderate level of agreement. The highest level of agreement was for BCS 3.5, which indicates good condition (Kappa 0.750, substantial agreement) with the least accurate overall score being BCS 5, indicating obesity (Kappa 0.400, fair agreement). 

## 4. Discussion

This study examined Traveller horse owners’ attitudes to horse welfare by focusing on BCS, a tool that is used to assess and monitor horse body condition. While the BCS tool was previously unknown to participants, the overall findings reflect a positive attitude to it, with participants prepared to use BCS as part of their routine management. The belief that all horse owning communities should use BCS is in agreement with the early work of Carroll and Huntington [32] and this is encouraging as it suggests that this Traveller group are receptive to new ideas that may improve the welfare of their horses. Of equal importance were the benefits of BCS highlighted by participants, with the horse’s health and welfare at the core of these views. These benefits included the identification of obesity and appropriate feeding management to help reduce the potentially adverse effects of obesity and are in agreement with previous studies citing body condition as a crucial factor in the development of appropriate dietary needs [48,49]. The evaluation and understanding of a horse’s body condition was considered a key factor in preventing future health problems. 

While agreeing that Carroll and Huntington’s anatomical descriptors were valid [32], the inclusion of two further areas, the chest and the hips, were suggested with claims that fat accumulates in these regions. While there are various instruments that use between five and eight distinct body regions to evaluate BCS in dairy herds [50,51,52], there are no known horse BCS tools that incorporate the additional areas suggested by participants. However, chest girth is measured when calculating body weight by the equation calculation [53]. In the current study, the hips were included within the assessment of the horse quarters. Nevertheless, this observation suggests that Traveller horse owners are aware of the anatomy of the horse and by recommending the inclusion of further areas in the BCS scale, the instrument’s accuracy may be improved. 

Visual appraisal was acknowledged as the on-going method of assessing horses’ body condition although visual appraisal alone is considered ineffective in determining BCS [31]. A likely explanation for participants’ viewpoints is that visual assessment has been the natural practice of this cultural group. With over 28% of Travellers having difficulty with literacy skills [44] and 55% of Travellers leaving school by age 15 years [54], it would seem fair to suggest that visual assessment is adopted to compensate for low literacy skills. The winter BCS of Assateague Island feral ponies (*n* = 36) was assessed by visual examination and found to be useful [55]. Given that Traveller horses live in herds, visual assessment could be used as a stand-alone method and is possibly more welfare compliant as horses can be assessed without the need for gathering or handling. 

Following the BCS practical assessment, a moderate level of agreement (kappa 0.45) was found for overall BCS scores between participants and the researcher, with these findings broadly similar to previous studies within the horse industry. Wyse et al. [34] reported a fair level of agreement, (kappa 0.40). Thus, the inclusion of the averaging system may have reduced the likelihood of scoring variation, allowing for more accurate assessment [46]. Participants also achieved a fair level of agreement when scoring the neck. As a “cresty” neck is associated with an increased risk of metabolic disorders [56], further training is recommended to improve score accuracy and reduce the risk of disease. BCS 5, indicating an obese horse, was the least accurate score for all body sections as well as for the overall assessment. Underestimating high BCS scores may lead to obesity and increased risk of laminitis [57] and infertility [58] in horses. Wyse et al. [34] also found less agreement at higher BCS scores and suggest that it is possibly due to participants’ reluctance to score their own horses as obese. Similarly, parents have been found to score their overweight child inaccurately [59]. It must be noted that in the current study, all horse owning participants scored at least one of their own horses.

While Traveller horse owners appear to have a moderate understanding of the application of BCS, inaccuracies were recorded. A possible reason for these inaccuracies is that horse owners had only a brief introduction to BCS and were not guided by the researcher during the scoring process. Further, locating the anatomical areas correctly and the presence of a winter coat may have reduced scoring accuracy. Pregnant mares may have been scored lower because of taut skin pulled over the ribs, although the middle body region (BCS 3) was where the most accuracy was found. These findings are in contrast to Goodwin [60], who found the least agreement for this body region, however, her sample size was much larger. A reason for the better performance of participants for the middle section may be revealed in the findings of the qualitative analysis. Participants showed a high level of awareness of how poor body condition may indicate other health related issues rather than lack of feed. Risk factors, such as grass-belly, parasites, and pregnancy, were identified and understood by participants. While there were inaccuracies in scoring, definitive conclusions cannot be derived from such a small sample. Therefore, the findings of BCS application should be considered with caution. By taking into account factors, such as breed, season, sample size, and experience [35,36,37], BCS could be improved as an educational tool to evaluate horse welfare. A limitation of the kappa statistics is that for high values of agreement, low values of kappa can be accepted and therefore interpretation may be considered too lenient. 

It became apparent that good health was almost universally regarded as an indicator for good welfare, a view reflecting Visser and Van Wijk-Jansens’ [3] study, where 99.6% of the participants also stated that good health relates to good welfare. In that study, respondents deemed a shiny coat, lively ears, and good performance as symbolic of good welfare while in this current study, references to a healthy horse included body condition, form, energy, weight, and coat and teeth condition. While good health is necessary for good welfare, other factors, as indicated by the five freedoms [23], need to be present in order to ensure good welfare. Generally, participants maintained that they were able to identify whether a horse was sick or healthy and identified appropriate behaviours associated with reduced horse well- being, although Lesimple and Hausberger [61] suggest that overexposure to certain behaviours may result in an underestimation of likely problems by owners/carers. They found a large discrepancy in responses between horse owners and caretakers in a study on horses’ stereotypic behaviours, with only 5% reporting any type of abnormal repetitive behaviours in horses compared to 37% of ethological observations. 

De-worming was considered to be crucial with the presence of parasites considered detrimental to a horse’s health, leading to weight loss, colic, and death [62]. This view is consistent with the findings of a survey conducted on worm control practices in equine establishments in Ireland whereby a high level of awareness was found on the need for worm control [63]. However, there was variation expressed on the required frequency of worm dosing in both the present study and O’Meara and Mulcahys’ study [63]. An explanation for this finding may be that as de-worming strategies have ranged in age from 18 to 54 years, it is likely that successive generations have been advised differently, thus leading to this lack of agreement. Further, with Travellers now leading a less nomadic life than in the past, it is likely that consultations with vets are more commonplace in Traveller culture. Therefore, de-worming approaches may also differ due to the increased opportunity for veterinary advice, which is supported by the fact that in this study, the younger age group were more likely to worm their horses according to veterinary advice. The belief that all horses should be de-wormed for prevention was mentioned, although Nielsen [64] proposes that this treat-all at regular intervals approach should be abandoned in favour of a more sustainable parasite control method. The Traveller tradition of keeping horses in herds may explain the view that group de-worming improves group welfare. Also noteworthy was that the practice of faecal egg count (FEC), which is cheap, easy to use, and an effective way of identifying the need for de-worming treatment, was not raised by participants in the present study. While the present sample is too small to draw conclusions that will be applicable to all Traveller horse owners, the need for a more sustainable equine parasite control programme is essential to help combat resistance to current de-worming practices.

Exercise was considered important to the horse’s physical and mental well-being and was accepted as standard for outdoor horses with structured exercise recommended for stabled horses. Management practices which result in lack of exercise are likely to result in the development of stereotypic behaviour, such as weaving or box walking [65,66,67,68]. 

The age cited to start exercise was approximately two years with handling beginning earlier. Exercising of two year olds horses is often considered inappropriate, with equine stakeholders in the study by Horseman et al. [69] study associating breaking horses in too young with Traveller horse culture. While the findings in this study may be an area of concern in terms of horse welfare, the youthfulness of the horse was also found to be a concern in the racing industry [70]. However, Standardbred harness racehorses and Thoroughbred flat racehorses that commenced training as 2-year olds were found to have a lengthier and more successful racing career than those who started training at a more advanced age [71,72]. Further, in the feral environment, foals are known to travel large distances with their dams with some foals as young as one week old travelling 7.3 km in a day [73]. Participants maintained that this age was appropriate for Traveller horses with habituation to traffic and behaviour management stated as reasons for starting the handling of horses at a young age. However, this belief is contrary to findings from a study by Mal et al. [74], which found that pre-weaning handling was found to have no influence on either foal manageability or learning performance. 

The subject of sulky racing arose during discussions over the exercise of horses. This activity is often criticised by the media and general public for being unregulated, unsafe, and detrimental to the horse’s welfare. Issues, such as road collisions involving sulkies and subsequent horse injuries and deaths, are cited in the media as animal welfare concerns although there is no empirical data to corroborate these reports. These views are often based on the negative stereotyping of Travellers, with these views extending to that of Traveller/Gypsy horse owners [75]. Consequently, there is a need for future research into this cultural activity from a Traveller’s perspective, thus allowing Travellers the opportunity to discuss sulky racing in the context of these criticisms rather than having to conform to the values and expectations of the settled community [21].

The need for social contact was recognised by participants and implemented in their management practices. This view is perhaps contradictory to many modern horse husbandry systems, where horses may be kept confined for up to 23 h/day to ensure optimal physical health with their need for social contact often neglected [76]. Further, participants maintained that horses form relationships and even have preferred companions within the herd, a view supported by Kimura [77], who found that mutual grooming relations were associated with bonds between individual horses. While horses are often kept in homogeneous groups to reduce the possibility of injury and to assist with management [78], the principal of keeping horses in herds was the accepted practice with horse owners in this study. Horses are social animals by nature [79] with isolation resulting in stress and stereotypical behaviours [80]. Management practices cited and adopted by the group demonstrate respect for the need for social interaction. The potential risk of bullying behaviour within the herd was recognised and considered as a possible welfare concern that may require addressing.

The overwhelming perception among participants is that horses have feelings, a view supported by the recent increase in the scientific study of an animal’s subjective experience [81]. However, the subjective nature of feelings and emotions is also the reason for the dismissal of animal sentience [82]. Proctor states that to achieve a positive change in attitudes and actions towards animals, it is essential to recognise animals as sentient [81]. The assertion by participants that fear is often the result of human interaction is strengthened by a study by Smith et al. [83], which found that horses’ reactions to negative human expressions were particularly strong This view is shared by Lansade et al. [84], who found that the quality of the human–animal interaction has a direct impact on the horse’s welfare. With attitudes found to be linked to the rate and nature of horse–human interactions [85], positive relationships can lead to mutual welfare benefits. The human–horse relationship was considered to be reciprocal, with benefits to participants’ own mental health cited and their connection to horses considered to be a positive outlet for their mental well-being. These benefits were possibly informed by participants’ own experiences. Both the physical and mental health benefits of animal-assisted therapies have been well documented [86,87,88]. Mental health issues can be a taboo subject within Traveller culture, with Traveller men in particular reluctant to discuss these issues [44], therefore, the subject of mental health and horse ownership within this group requires further investigation. Further, horses are often regarded as friends by Travellers rather than as possessions [89]. 

There was a high degree of consensus on the importance of the expression of natural behaviour. The Traveller horse owners’ perspective supports the view that horses should spend most of their time outdoors, which corresponds with the findings of a study by Jørgensen et al. [90], who found that most horses had a preference to remain outdoors irrespective of the weather. This approach differs to a minority of leisure horses in the UK who were reported to have had no access to pasture in the week previous to the completion of a survey by their carers [91].

By limiting the expression of natural behaviours, stereotypies may be observed [92]. Therefore, allowing horses to remain outdoors is the most effective way of preventing stereotypical behaviours [93], consequently improving welfare [94]. In their natural environment, feral horses graze approximately 18 hours a day [95] and will roam up to 80 km a day [96]. In the same way, constant access to grazing with additional feed when necessary was the view expressed by participants. The practice of seasonal feeding patterns is supported by the body condition scores of horses assessed in this study, with the scores obtained indicative of fat storage as a buffer against feed shortage in the winter. 

The dominant view of participants is that rugs are only required in exceptional circumstances. This differs from Goodwin’s finding, where more than 86% of participants from livery yards in Scotland were found to consistently rug their horses during winter [60]. Further, participants stated that the settled community often questions Traveller horse owners’ rug practices, believing that un-rugged horses are neglected. This may explain why some participants have adopted the practice of rugging. However, according to Jørgensen et al. [90], horses are able to sustain a suitable internal body temperature in temperatures varying from −30 °C to 30 °C. Shelter along with sufficient food was found to be adequate to keep horses warm during the winter [ibid], a finding echoing the views of the majority of participants. 

Opinions were divided on the issue of horse breeding with participants recommending either an absolute ban or breeding control with most stressing the importance of resource availability and provision to ensure optimal welfare. There was an overall agreement amongst equine stakeholders in the study by Collins et al. [5] that the production of horses had exceeded demand in Ireland. With horses no longer in great demand, indiscriminate breeding is considered to compromise their welfare status [97]. Indiscriminate breeding is often associated with Traveller horse owners, a viewpoint reported as recently as December 2018 by a senior scientific equine advisor at the Royal Society for the Prevention of Cruelty to Animals in The Sunday Telegraph. However, Horseman et al. [69], in their study on welfare issues facing horses in Great Britain, found that equine stakeholders assigned the problem of overbreeding to the racing sector. In this study, participants’ perception that welfare issues exist throughout the industry, including within their own community, is consistent with the findings from Collins et al. [5], which demonstrated that welfare concerns are not confined to any one group. 

One of the major barriers to good horse welfare that was identified, and which is unique to Travellers, is the lack of availability of land on which to keep their horses. This view is supported by Fritzsche’s [16] findings on landowners’ reluctance to lease land to Travellers. Current policy simply addresses the symptoms of this issue through the Control of Horses act [14] rather than looking at the root causes. However, one possible solution is the provision of land and support through horse projects. Although this was a very small sample from one horse project, participants commented positively on the impact that it had had on their lives. Horse projects may help to create a supportive and sustainable solution to Traveller equine issues [12], while also facilitating Travellers’ tradition of horse ownership. Further research is required in order to fully evaluate the impact of horse projects on both horse welfare and human well-being.

## 5. Conclusions

The findings in this study provide us with a unique snapshot of Traveller horse owners’ attitudes to BCS and perceptions of horse welfare as well as an assessment of the application of the BCS tool for accuracy and ease of use by this group. Themes were found to be particularly rich in relation to participants’ understanding of the natural behaviour and natural environment of the horse and its application in their management practices, an approach currently gaining recognition both within equine science and the general horse owning community. Further investigation of these findings with larger sample groups is recommended as this would be advantageous to both the research process and the Traveller community To conclude, participants in this study expressed a good knowledge and understanding of horse management, and perhaps more importantly, positive attitudes to horse welfare. These findings indicate that, given the appropriate support and access to resources, there is the potential for a good level of welfare in horses owned and managed by this cultural group.

## Figures and Tables

**Table 1:** Questions used in the interviews and discussion groups.

**Body Condition Scoring (BCS)**
Are you familiar with body condition scoring?
What is your view on the looking/feeling of body fat on a horse to assess its condition?
Do you think BCS works and can you use it on all horses?
How do you tell whether what you are feeling is fat or muscle?
What are the benefits of using BCS?
Why does it matter to know the body condition of your horse?
Would you include any further parts of the horse’s body in BCS?
How often should BCS be used?
Who should use BCS and would you use BCS in the management of your horses?
**Feeding**
Is body condition a good guide of how suitable a horse’s diet is?
Do you believe that horses should have constant access to grazing?
What is the difference between a horse having a ‘grass belly’ and being very fat/obese?
**Health**
By understanding your horse’s body condition, do you think it will help prevent health problems?
Can you tell if a horse is healthy by looking at it?
What does a healthy horse look like?
Can you tell if a horse is sick? If so, how?
Do you think horses need rugs in the winter?
How important is it to worm horses?
Do you think a healthy horse is a sign of good welfare?
**Exercise**
How important is exercise for a horse?
What sort of exercise do you use?
At what age do you consider it suitable to begin exercising a horse?
**Sentience**
Do you believe that horses have feelings?
**Social**
How do you think horses prefer to live (groups, individually)? Why?
Do you think that horses develop bonds with other horses and their owners?
**Breeding**
In the current economic climate, what is your view on the breeding of horses?
Welfare
Do you believe that there are horse welfare problems throughout the horse sector?
**Difficulties**
What are some of the difficulties of horse ownership for Travellers?
What are your thoughts on how things could be improved for Traveller horse owners?
Is there anything else that you would like to say that you feel is important?

**Table 2:** Revised descriptors of the BCS chart (adapted from Carroll C.L. and Huntington P.J. [31]).

Score	Image	Description
**Score 0**	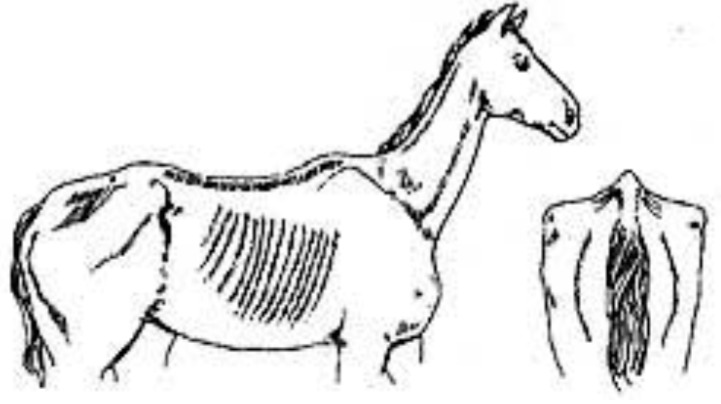	**Neck/Shoulders**: Fat not visible; **Middle**: Ribs seen easily, backbone sticking out; **Hind quarters**: Very hollow.
**Very Thin**		
**Score 1**	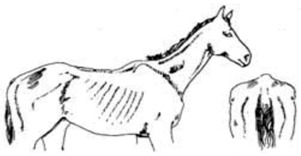	**Neck/Shoulders**: Narrow and slack; **Middle**: Ribs easily visible, backbone sticking out; **Hind quarters**: Hollow.
**Thin**		
**Score 2**	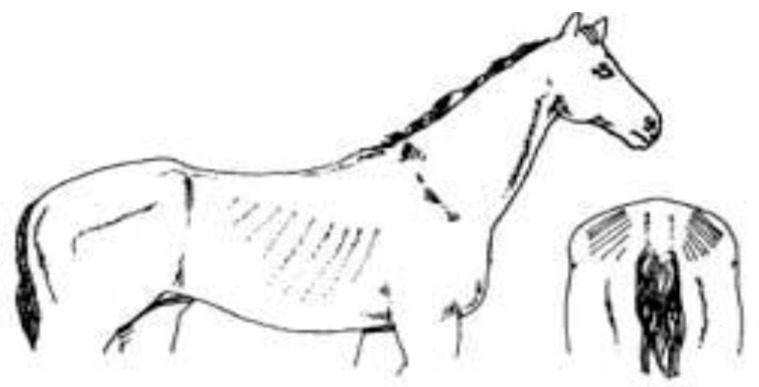	**Neck/Shoulders**: Narrow but firm neck; **Middle**: Ribs just visible; **Hind quarters**: Flat rump at both sides.
**Moderate**		
**Score 3**	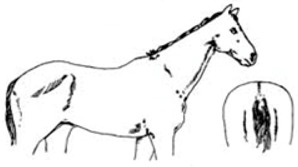	**Neck/Shoulders**: No crest visible; **Middle**: Ribs just covered; **Hind quarters**: Rounded Rump.
**Good**		
**Score 4**	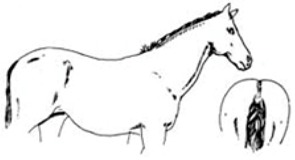	**Neck/Shoulders**: Slight crest visible; **Middle**: Ribs and pelvis—covered; **Hind quarters**: Rump well rounded.
**Fat**		
**Score 5**	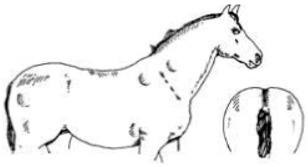	**Neck/Shoulders**: Fat pads visible; **Middle**: Visible fat pads on back; **Hind quarters**: Visible fat pads at either side of tail.
**Very Fat**		

**Table 3:** Level of agreement between participants/researcher’s BCS.

Section Scored	Agreement Percentage	95% Confidence Level	Cohen’s Kappa Statistic	Level of Agreement
Overall	66,67%	34.89, 90.08	0.445	Moderate
Neck	50.0%	21.09, 78.91	0.345	Fair
Middle	66.67%	34.89, 90.08	0.445	Moderate
Hindquarters	41.67%	15.17, 72.33	0.207	Slight
